# The availability of jobs in the biopharmaceutical industry is 45-fold greater for hematology oncology than medical specialties

**DOI:** 10.1038/bcj.2017.91

**Published:** 2017-09-15

**Authors:** V Kaestner, V Prasad

**Affiliations:** 1 Division of Hematology and Oncology, Knight Cancer Institute, Oregon Health and Science University, Portland, OR, USA; 2 Division of Hematology and Medical Oncology, Knight Cancer Institute, Oregon Health and Science University, Portland, OR, USA; 3 Department of Preventive Medicine and Public Health, Oregon Health and Science University, Portland, OR, USA; 4 Center for Health Care Ethics, Oregon Health and Science University, Portland, OR, USA

The role of the biopharmaceutical industry in medicine has been the subject of intense study and debate. Often, the industry’s influence is measured by rates of financial conflict of interest among medical doctors. Recent studies show that 48% (449 864/933 295) of the United States-based physicians received a general payment from the biopharmaceutical industry in the year 2015.^1^ A general payment is one made to a physician personally, and may be contrasted with a research payment made to their institution. In certain specialties, such as Neurosurgery and Medical Oncology, up to 66.1%^1^ and 63%^2^ of physicians receive general payments, respectively.

Rates of personal financial conflict may not fully measure or capture the role of the biopharmaceutical industry in that discipline. Some fields may have disproportionate job opportunities for specialists within the industry. The opportunity for industry employment may constitute another metric of industry influence. As example, high subsequent industry employment within the biomedical industry was recently demonstrated for oncology medical reviewers at the US Food and Drug Administration.^3^

Using two widely used job posting sites, we sought to identify the prevalence of industry-related jobs available to physicians, and whether medical oncology had more industry postings than general medical disciplines.

We identified consecutive jobs posted on the New England Journal of Medicine (NEJM) Career Center (http://www.nejmcareercenter.org/searchjobs/) and the American Society of Clinical Oncology (ASCO) Career Center (https://careercenter.asco.org/jobs/). The search and analysis was conducted between 18 May 2017 and 19 June 2017, and we included consecutive jobs posted between 10 March 2017 and 19 June 2017 with no filter.

Job postings were included in our analysis if they required or preferred candidates with an MD or DO degree. We chose this inclusion criterion because we were concerned with career opportunities for physicians. For each post we extracted: the job title, specialty of job, company/university/group, industry/academic/private, salary, required or preferred traits, and posting date.

A general search of the NEJM Career center yielded a sample of 365 included in our analysis. A general search of the ASCO Career center yielded a sample of 346 jobs included in our analysis.

Of 365 job posts in the NEJM Career Center included in our analysis, 148/365 (41%) were academia affiliated, 2/365 (0.5%) were industry affiliated and 215/365 (59%) were affiliated with a private practice. In contrast, of 346 job posts in the ASCO Career Center included in our analysis, 116/346 (34%) were academia affiliated, 78/346 (23%) were industry affiliated and 152/346 (44%) were affiliated with a private practice. A breakdown of these posts from each site and *P*-values for comparison are shown in Table 1.

In short, we find a 45-fold (4500%) increased likelihood of an industry job posting directed at oncologists rather than general physicians. This phenomenon may provide additional information about the oversized role of the industry in oncology. 48% of all the US physicians take general payments from biopharmaceutical companies, in contrast to 63% of the US medical oncologists. Yet, our analysis of job posts shows a far greater difference (45-fold increase) and may reflect a different, yet widespread, form of industry influence in oncology.

## Figures and Tables

**Table 1:** Breakdown of types of job postings requiring or preferring an MD/DO in each career center

	*Academic no. (% of total)*	*Industry no. (% of total)*	*Private no. (% of total)*	*Total*
NEJM	148 (41%)	2 (0.5%)	215 (59%)	365
ASCO	116 (33.5%)	78 (22.5%)	152 (44%)	346
*P*-value for comparison	*P*=0.054	*P*<0.001	*P*=0.001	

Abbreviations: ASCO, American Society of Clinical Oncology; NEJM, New England Journal of Medicine.
