# The availability of jobs in the biopharmaceutical industry is 45-fold greater for hematology oncology than medical specialties

**DOI:** 10.1038/bcj.2017.99

**Published:** 2017-11-03

**Authors:** V Kaestner, V Prasad

**Correction to:** *Blood Cancer Journal* (2017) **7**, e609; doi:10.1038/bcj.2017.91. Published online 15 September 2017

In our paper, the title refers to job advertisements (as articulated clearly in the text) and not jobs *per se*. The title should read ‘Job postings for biopharmaceutical industry in medical journals is 45-fold greater for hematology oncology than medical specialties.’ We also wish to clarify that 45-fold increase corresponds to 4400% increase (not 4500% as stated in the paper). Finally, we wish to revise the concluding sentence of the paper to read, ‘Yet, our analysis of job posts reflects a different, yet widespread, form of industry influence in oncology.’ Without formal analysis, we recognize that we cannot compare the relative influence of this finding to the influence of financial conflict of interest.

We also wish to disclose a potential conflict of interest: VP receives royalties from his book Ending Medical Reversal.

